# Single-Cell RNA Sequencing Reveals an Atlas of Hezuo Pig Testis Cells

**DOI:** 10.3390/ijms25189786

**Published:** 2024-09-10

**Authors:** Zunqiang Yan, Pengfei Wang, Qiaoli Yang, Shuangbao Gun

**Affiliations:** College of Animal Science and Technology, Gansu Agricultural University, Lanzhou 730070, China; yanzq@gsau.edu.cn (Z.Y.); wangpf815@163.com (P.W.)

**Keywords:** Hezuo pig, scRNA-seq, testis, spermatogenesis, germ cell

## Abstract

Spermatogenesis is a complex biological process crucial for male reproduction and is characterized by intricate interactions between testicular somatic cells and germ cells. Due to the cellular heterogeneity of the testes, investigating different cell types across developmental stages has been challenging. Single-cell RNA sequencing (scRNA-seq) has emerged as a valuable approach for addressing this limitation. Here, we conducted an unbiased transcriptomic study of spermatogenesis in sexually mature 4-month-old Hezuo pigs using 10× Genomics-based scRNA-seq. A total of 16,082 cells were collected from Hezuo pig testes, including germ cells (spermatogonia (SPG), spermatocytes (SPCs), spermatids (SPTs), and sperm (SP)) and somatic cells (Sertoli cells (SCs), Leydig cells (LCs), myoid cells (MCs), endothelial cells (ECs), and natural killer (NK) cells/macrophages). Pseudo-time analysis revealed that LCs and MCs originated from common progenitors in the Hezuo pig. Functional enrichment analysis indicated that the differentially expressed genes (DEGs) in the different types of testicular germ cells were enriched in the PI3K–AKT, Wnt, HIF-1, and adherens junction signaling pathways, while the DEGs in testicular somatic cells were enriched in ECM–receptor interaction and antigen processing and presentation. Moreover, genes related to spermatogenesis, male gamete generation, sperm part, sperm flagellum, and peptide biosynthesis were expressed throughout spermatogenesis. Using immunohistochemistry, we verified several stage-specific marker genes (such as *UCHL1*, *WT1*, *SOX9*, and *ACTA2*) for SPG, SCs, and MCs. By exploring the changes in the transcription patterns of various cell types during spermatogenesis, our study provided novel insights into spermatogenesis and testicular cells in the Hezuo pig, thereby laying the foundation for the breeding and preservation of this breed.

## 1. Introduction

The Hezuo pig (also called the Juema pig) is an indigenous breed to Gansu Province. It is found in the northern area of the Tibetan Plateau and adjacent alpine and subalpine regions, at an altitude of 2500–3800 m, where there is no absolute frost-free period [1,2,3]. Hezuo pigs have dense fluff, a compact body structure, and a relatively small skin surface area per unit of body weight, characteristics that allow them to adapt well to harsh environments such as extreme cold, low oxygen (hypoxia), and strong ultraviolet radiation [3,4,5]. In addition to providing meat for the area and representing a vital source of income for local herders, they are also important for high-quality socioeconomic development, pasture ecosystem maintenance, and domestic animal biodiversity conservation on the Tibetan Plateau [6,7,8]. The Hezuo pig has lower reproductive performance than other pig breeds, such as Taihu pigs [3,4]. Reproductive improvement in Hezuo pigs, particularly males, is the key to improving the production performance of this breed. Further research on Hezuo pig testes could enhance its reproductive efficiency. In our previous work, we found that Hezuo pigs displayed precocious puberty compared to Landraces. We identified several differentially expressed lncRNAs, such as *LOC102166140* and *MSTRG.15011.2*, that were associated with spermatogenesis and were enriched in ECM–receptor interaction and the PI3K–Akt, TGF-beta, and MAPK signaling pathways [4]. We also investigated the testes of Hezuo pigs before and after sexual maturity and identified several differentially expressed miRNAs (such as ssc-miR-29b and ssc-miR-370) that may be related to precocious puberty [9].

The testes are essential organs in the male reproductive system, primarily composed of spermatogenic tubules and testicular mesenchyme [10]. The spermatogenic tubules are composed of connective tissue fibers, basement membranes, and stratified germinal epithelium from outside to inside, and the germinal epithelium is primarily made up of spermatogenic cells (including spermatogonia (SPG), primary spermatocytes (SPCs), secondary spermatocytes (SPCs), spermatids (SPTs), and sperm (SP)) and Sertoli cells (SCs) [11,12]. The testicular mesenchyme, on the other hand, mainly contains Leydig cells (LCs), myoid cells (MCs), and macrophages [13]. Extensive evidence indicates that the testis perform both exocrine and endocrine functions [14]. The exocrine function involves the production of highly specialized gametes, known as SP, which are stored in the epididymis [15,16,17]. While endocrine function involves androgen (mostly testosterone) secretion by LCs, which is crucial for maintaining male secondary sex characteristics as well as promoting male testis development and spermatogenesis [10,18]. These observations underscore that spermatogenesis is a comprehensive and precisely regulated biological process, requiring the coordinated interaction of various cell types under the influence of numerous factors, including hormones, paracrine signals, and epigenetic regulators [12,19]. Therefore, maintaining the physiological integrity of the testes is essential for effective sperm production [20,21]. However, the heterogeneity of germ and somatic cells within the testes poses significant challenges in studying the various cell types at different developmental stages and assessing gene expression within specific cell populations [22].

It has become possible to obtain thousands of independent features per cell using the 10× Genomics scRNA-seq platform, thereby providing ultra-high-resolution transcriptomes of tissues and organs [23,24,25,26]. Many studies have employed this unbiased approach to investigate spermatogenesis in a variety of animals, including the mouse [27], buffalo [28], yak [19,29], goat [30,31], pig [32,33], sheep [34], giant panda [35], and humans [36]. For example, Yi et al. undertook a scRNA-seq analysis of the transcriptome of the testes of giant pandas and obtained 5431 cells, among which eight cell types could be identified, including six types of somatic cells and two types of germ cells [35]. Huang et al. explored dynamic gene expression roadmaps relating to self-renewal and differentiation of germ and somatic cell development in the testes of prepubertal (3 months old) and pubertal (24 months old) buffalo, describing the key transcriptional changes occurring during the entire process of spermatogenesis [28]. Wang et al. [29] studied spermatogenesis in sexually mature (4-year-old) yaks and identified six somatic cell and various germ cell types, as well as many differentially expressed genes (DEGs) that were related to spermatogenesis via the MAPK, cAMP, ECM–receptor interaction, and PI3K–Akt signaling pathways in yak testes. Additionally, two genes showing cell-specific expression (*BMX* for spermatogonial stem cells and *CRYAB* for SCs) were identified and verified [19].

However, research relating to the process of gametogenesis in the Hezuo pig is very limited. Here, using the 10× Genomics scRNA-seq platform, we explored the gene expression profiles of cells obtained from the testis of a healthy, sexually mature Hezuo pig. We established expression profiles for 16,082 cells and identified eight testicular cell types, including four types of germ cells and five types of somatic cells. The differentially expressed genes (DEGs) in each cell type were screened, and their functions (GO terms and KEGG signaling pathways) were analyzed. This study greatly extends the knowledge of the mechanisms involved in the regulation of testicular development and spermatogenesis in male Hezuo pigs, laying the foundation for an in-depth study on the breeding and preservation of this valuable breed.

## 2. Results

### 2.1. Overview of the scRNA-Seq Analysis of Testicular Cells

**Figure 1:**
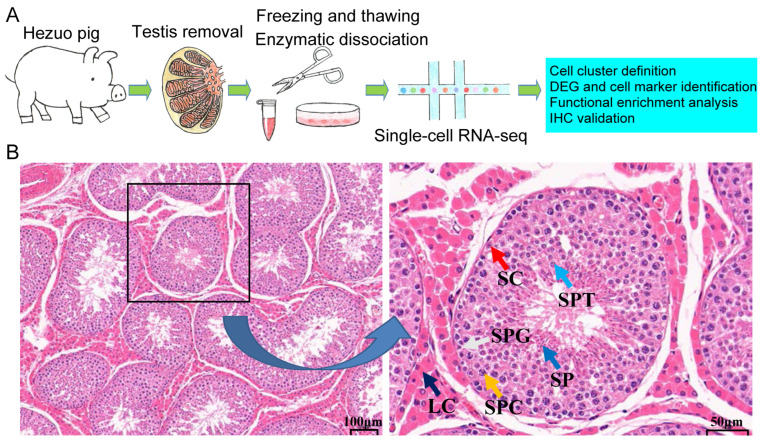
Schedule of the scRNA-seq analysis of testicular cells of the Hezuo pig. (**A**) A schematic diagram of the experimental workflow for the scRNA-seq analysis of testicular cells. First, testicular samples were obtained from a healthy, sexually mature male Hezuo pig via surgical methods by a veterinarian. Secondly, the testis were cut into small fragments and cryopreserved in liquid nitrogen. Thirdly, following thawing, the testis fragments were subjected to enzymatic dissociation followed by scRNA-seq. Finally, cell cluster definition, differentially expressed gene (DEG) and cell marker identification, and functional enrichment analysis were conducted based on the sequencing data. Additionally, testicular cell markers were validated by immunohistochemistry on testicular sections. (**B**) Histological observation of testicular sections. spermatogonia (SPG), spermatocytes (SPCs), spermatids (SPTs), sperm (SP), Sertoli cells (SCs), Leydig cells (LCs). Left: ×100 magnification, scale bar = 100 μm; right: ×400 magnification, scale bar = 50 μm.

To identify the various cell types and related marker genes in the testes of Hezuo pigs, we conducted a scRNA-seq analysis on cells isolated from frozen testicular tissue collected from a healthy male Hezuo pig (Figure 1A). Hematoxylin and eosin (H&E) staining indicated the presence of different types of spermatogenic cells (including SPG, SPCs, SPTs, and SP) and somatic cells (such as SCs and LCs) in the Hezuo pig testis used for scRNA-seq analysis (Figure 1B).

**Figure 2:**
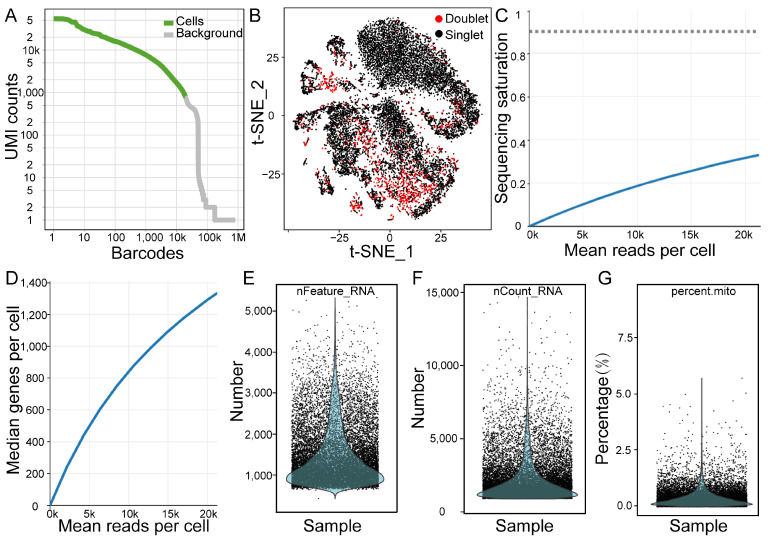
Data quality. (**A**) A map of effective cell identification. The abscissa is the number of barcode sequences, and the ordinate is the number of unique molecular identifiers (UMIs). The green line of the barcode corresponds to the effective cell count, and the gray line represents the background noise. (**B**) t-distributed stochastic neighbor embedding (t-SNE) plot showing the cellular distribution. Each point in the figure represents a cell. (**C**) The map of sequencing saturation. (**D**) The median genes per cell as a function of downsampled sequencing depth in mean reads per cell, up to the observed sequencing depth. (**E**) The distribution of the number of genes detected. (**F**) The distribution of the total number of unique molecular identifiers (UMIs) detected. (**G**) The percentage distribution of mitochondrial genes expressed in individual cells.

Following the digestion of the testicular tissue samples, the viability of the cells was found to be approximately 95%. The cells were used to construct a single-cell library, which was subsequently sequenced using the 10× Genomics platform. A total of 18,783 cells were obtained (Table S1), resulting in the retention of 16,082 effective cells (Figure 2A) according to specific filtration criteria, including the deletion of doublets (Figure 2B). A total of 335.8 Mb of reads were generated from the libraries, and the sequencing saturation rate was 33.00% (Figure 2C). A total of 24,760 genes were detected in all the cells, with a median of approximately 1336 genes per cell (Figure 2D). The computed cell-level quality metrics for the data are displayed in Figure 2E–G and Table S2. For example, each cell had 1779 unique molecular identifiers (UMIs), and the mitochondrial UMI ratio was approximately 0.35%.

### 2.2. Clustering Analysis and Cell Type Identification

**Figure 3:**
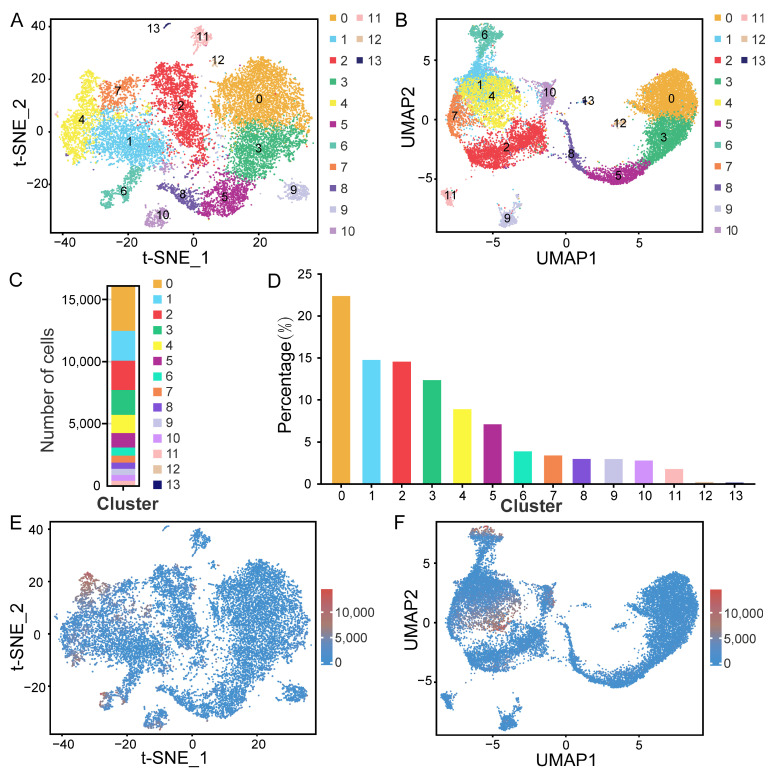
Single-cell transcriptome profiling and cluster identification in testicular cells. (**A**) t-distributed stochastic neighbor embedding (t-SNE) and (**B**) uniform manifold approximation and projection (UMAP) plots showing the 10× Genomics profile of unselected spermatogenic cells. Cell clusters are distinguished by color. (**C**) Classification stacking diagram showing the number of cells in each of the 14 clusters. (**D**) Histogram showing the proportion of cells in each of the 14 clusters. (**E**) t-SNE and (**F**) UMAP plots showing the transcript expression levels based on unique molecular identifiers (UMIs).

**Figure 4:**
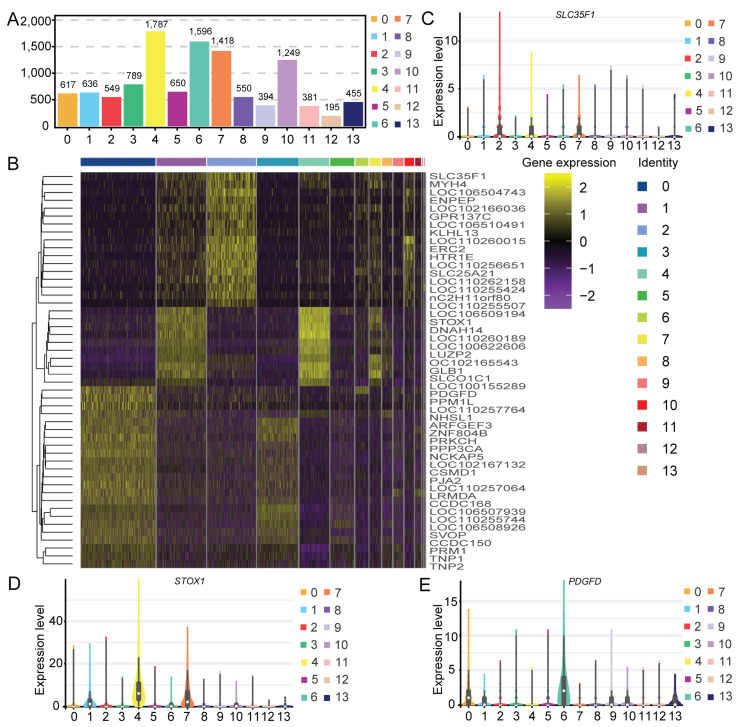
Identification of differentially expressed genes (DEGs). (**A**) The number of DEGs in each cluster. (**B**) A heatmap of the top 50 DEGs among the clusters. In the upper panel, the cell clusters are differentiated by color. The colors from red to blue represent the expression level from high to low, respectively. (**C**–**E**) Violin plots illustrating the expression patterns of three selected DEGs in each cell cluster.

To overcome the extensive technical noise associated with single genes in scRNA-seq data, the scRNA-seq expression matrix was subjected to t-distributed stochastic neighbor embedding (t-SNE) and uniform manifold approximation and projection (UMAP) analyses. Based on gene expression patterns, the 16,082 cells were divided into 14 clusters (9 germ cell and 5 somatic cell clusters) via t-SNE analysis (Figure 3A) and UMAP analysis (Figure 3B). The number and the respective percentage of cells in each cluster are displayed in Figure 3C and Figure 3D, respectively. Cluster 0 had the greatest number of cells (3613), accounting for 22.48% of the total, while cluster 13 had the fewest cells (45), accounting for 0.28%. And, cluster 12 had the 58 cells, accounting for 0.36%. Moreover, most cells had low transcript expression levels (Figure 3E,F). We also detected between 195 and 1787 DEGs in each cell cluster (Figure 4A). The top 50 DEGs among all the clusters are displayed in Figure 4B. Three DEGs (*SLC35F1*, *STOX1*, and *PDGFD*) were randomly selected for the validation of their expression levels in all the clusters (Figure 4C–E). As expected, these DEGs showed the highest expression levels in respective clusters.

**Figure 5:**
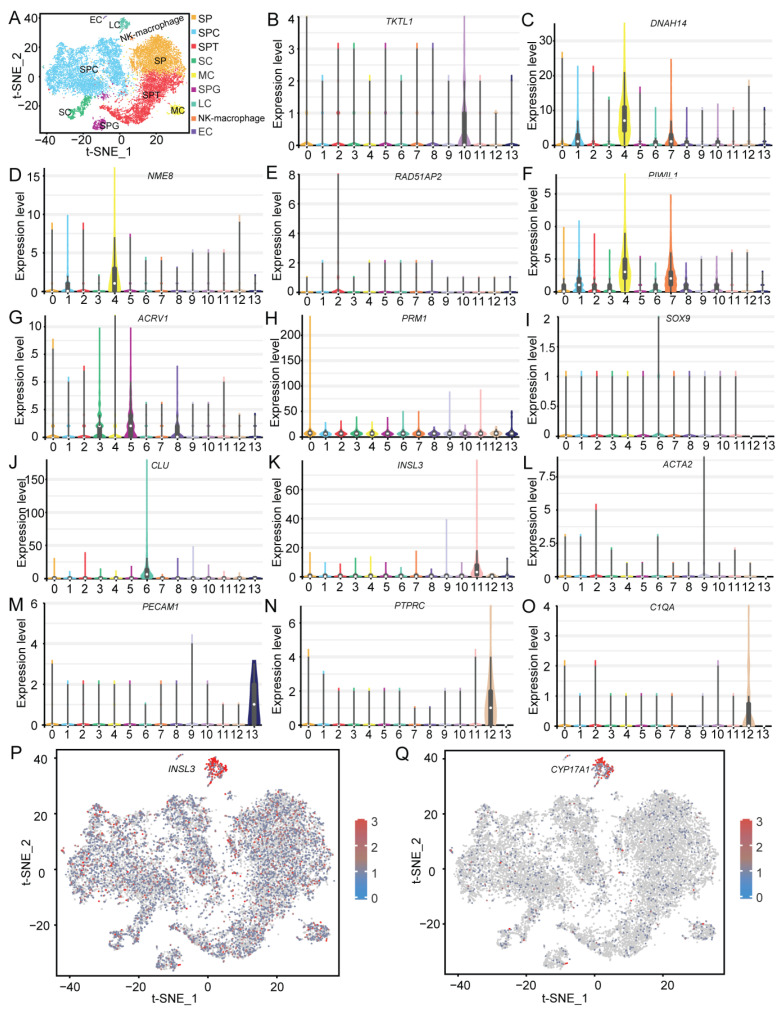
Cell type identification in testicular tissue. (**A**) t-distributed stochastic neighbor embedding (t-SNE) plot of the results of cell type identification. (**B**–**O**) Violin plots of different cell type-specific gene expression patterns across different clusters. (**P**,**Q**) t-SNE plots of selected marker gene expression across all clusters.

Given the total lack of availability of markers for male Hezuo pig germ cells, the known corresponding universal marker genes in humans, mice, and other animals were used to identify the cell types in each cluster based on the genes expressed in each cluster. Using this approach, we identified nine cell types in Hezuo pig testicular tissue (Figure 5A). The cells in cluster 10 were found to express the SPG marker genes *TKTL1* and *UCHL1* (Figure 5B and Figure S1A). The cells in clusters 1, 2, 4, and 7 expressed the SPC marker genes *DNAH14*, *NME8*, *RAD51AP2*, *PIWIL1*, *SLC9B1*, *DNAH12*, and *SYCP1* (Figure 5C–F and Figure S1B–D). The cells in clusters 3, 5, and 8 expressed the SPT marker genes *ACRV1*, *TEX29*, *ACR*, and *SPACA1* (Figure 5G and Figure S1E–G). The cells in cluster 0 expressed the SP marker genes *PRM1* and *SPTBN4* (Figure 5H and Figure S1H). The cells in cluster 6 expressed the SC marker genes *SOX9*, *CLU*, *WT1*, and *PRND* (Figure 5I,J and Figure S1I,J). The cells in cluster 11 expressed the LC marker genes *INSL3* and *CYP17A1* (Figure 5K and Figure S1K). The cells in cluster 9 expressed the MC marker genes *ACTA2*, *DCN*, *PDGFRA*, and *PTCH1* (Figure 5L and Figure S1L–N). The cells in cluster 13 expressed the EC marker genes *PECAM1* and *VWF* (Figure 5M and Figure S1O). The cells in cluster 12 expressed the NK cells/macrophage marker genes *PTPRC*, *C1QA*, *IL7R*, *CD74*, and *CSF1R* (Figure 5N,O, and Figure S1P–W).

A bubble chart of the expression patterns of these marker genes showed that all clusters were clearly separated (Figure S2). A heatmap of these marker genes demonstrated that specific genes clustered together to some extent (Figure S3). For example, the SC marker genes *SOX9* and *WT1* were clustered together; similarly, the SPT marker genes *ACRV1*, *TEX29*, *ACR*, and *SPACA1* were also clustered together. In addition, all these selected marker genes were expressed across all clusters based on t-SNE plots (Figure 5P,Q and Figure S4). For example, *INSL3* and *CYP17A1*, which are related to a high level of testosterone production, exhibited higher expression levels in LCs (cluster 11) than in the other clusters.

### 2.3. MCs and LCs Developed and Differentiated from the Same Progenitor Cells

**Figure 6:**
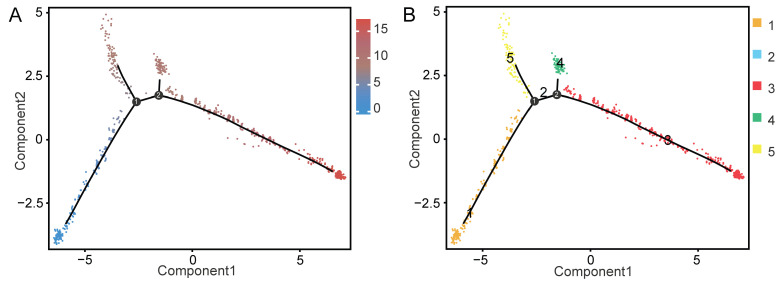
Pseudo-time analysis of myoid cells (MCs) and Leydig cells (LCs). Pseudo-time information (**A**) and differentiation status information (**B**) for the pseudo-time trajectory of clusters 9 and 11 predicted a common progenitor for the myoid and Leydig lineages. Pseudo-time information represents the developmental period, with the smaller the pseudo-time value, the earlier the developmental period. Different colors represent different differentiation states.

Cluster analysis with dimensionality reduction indicated that LCs were located in cluster 11 and MCs in cluster 9. Guo et al. [37] and Wang et al. [19] found that, in animal testes, MCs and LCs developed from the same progenitor cells. To investigate whether this was also the case for Hezuo pigs, a Monocle pseudo-time analysis was performed on clusters 9 and 11. The results demonstrated that MCs and LCs differentiated from common progenitor cells (Figure 6A,B).

### 2.4. Functional Enrichment Analysis of Testicular Somatic Cells

**Figure 7:**
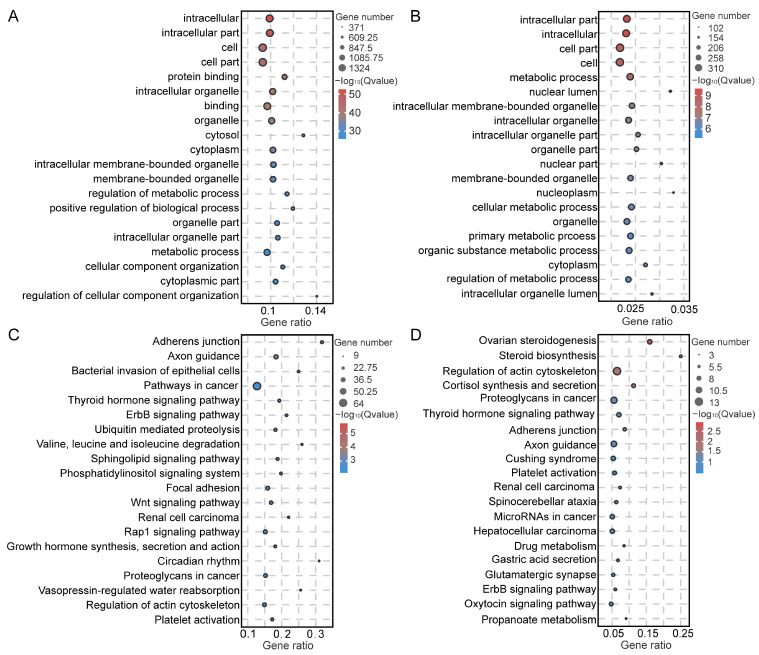
Functional enrichment analysis of SCs and LCs. The top 20 GO terms associated with the DEGs in SCs (**A**) and LCs (**B**). The top 20 KEGG pathways related to the DEGs in SCs (**C**) and LCs (**D**).

Testicular somatic cells play an important role in testicular development and spermatogenesis. Thus, we conducted GO-term functional enrichment analysis for the five types of somatic testicular cells identified. The results revealed that the DEGs in SCs were significantly enriched in terms such as regulation of metabolic process, positive regulation of biological process, and regulation of cellular component organization in the biological process category; in the cellular component category, they were enriched in terms such as intracellular part, intracellular organelle, cell part, and cytosol; and in the molecular function category, they were enriched in protein binding and enzyme binding (Figure 7A and Table S3). The DEGs in LCs were enriched in metabolic process and organic substance metabolic process in the biological process category; intracellular part, intracellular membrane-bounded organelle, and intracellular organelle part in the cellular component category; and protein binding and enzyme activator activity in the molecular function category (Figure 7B and Table S4). In MCs, the DEGs were significantly enriched in terms such as single-multicellular organism process, developmental process, and regulation of cell migration in the biological process category; extracellular matrix component and basement membrane in the cellular component category; and protein binding and extracellular matrix structural constituent in the molecular function category (Figure S5A and Table S5). The DEGs in ECs were significantly enriched in system development, biological adhesion, and cell migration in the biological process category; in the cellular component category, they were enriched in the terms cell, cell junction, and adherens junction; and in the molecular function category, they were enriched in protein binding and protein domain specific binding (Figure S5B and Table S6). The DEGs in NK cells/macrophages were primarily involved in immune response, activation of immune response, response to cytokine, and immune system processes in the biological process category; intracellular part, cell part, and intracellular non-membrane-bounded organelle in the cellular component category; and binding and enzyme binding in the molecular function category (Figure S5C and Table S7).

Additionally, we performed KEGG functional enrichment analysis for SCs, LCs, MCs, ECs, and NK cells/macrophages. The DEGs in SCs were significantly enriched in the thyroid hormone and Wnt signaling pathways (Figure 7C and Table S3). The DEGs in LCs were mainly involved in signaling pathways associated with steroid biosynthesis and cortisol synthesis and secretion (Figure 7D and Table S4). The DEGs in MCs were mainly involved in the PI3K–AKT and vascular smooth muscle contraction signaling pathways (Figure S5D and Table S5). The DEGs in ECs were greatly enriched in the adherens junction and ECM–receptor interaction signaling pathways (Figure S5E and Table S6). Finally, the DEGs in NK cells/macrophages displayed significant enrichment in the antigen processing and presentation, allograft rejection, and endocytosis signaling pathways (Figure S5F and Table S7).

### 2.5. Functional Enrichment Analysis of Testicular Germ Cells

**Figure 8:**
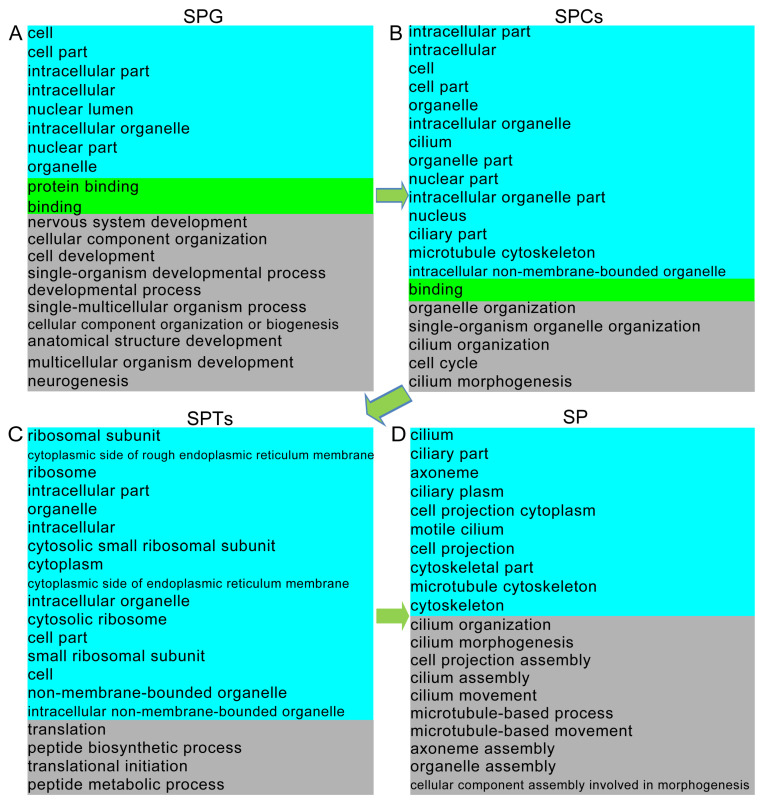
GO term enrichment analysis of the DEGs in (**A**) SPG, (**B**) SPCs, (**C**) SPTs, and (**D**) SP.

Spermatogenesis involves complex and systematic changes in spermatogenic cells. To explore the differences between the various germ cell types identified, the DEGs of each germ cell cluster were annotated against the GO and KEGG databases. We found that the DEGs related to protein binding, cell development, developmental process, cell part, spermatogenesis, male gamete generation, cytoskeleton, sperm part, gamete generation, sperm flagellum, peptide biosynthetic process, and other functions were displayed from SPG to SP throughout the overall spermatogenic process (Figure 8A–D and Tables S8–S11).

The DEGs in SPG were mainly enriched in the adherens junction, PI3K–Akt, Wnt, and cell cycle signaling pathways (Figure S6A and Table S8). The DEGs in SPCs were markedly enriched in the regulation of the actin cytoskeleton and the PI3K–Akt signaling pathways (Figure S6B and Table S9). In SPTs, the DEGs were significantly associated with the phosphatidylinositol signaling system and the Ras and purine metabolism signaling pathways (Figure S6C and Table S10). Additionally, the DEGs in SP were significantly related to signaling pathways involved in glycerophospholipid metabolism, ubiquitin-mediated proteolysis, necroptosis, endocytosis, vitamin digestion, and absorption (Figure S6D and Table S11).

### 2.6. Characterization of Testicular Cells

**Figure 9:**
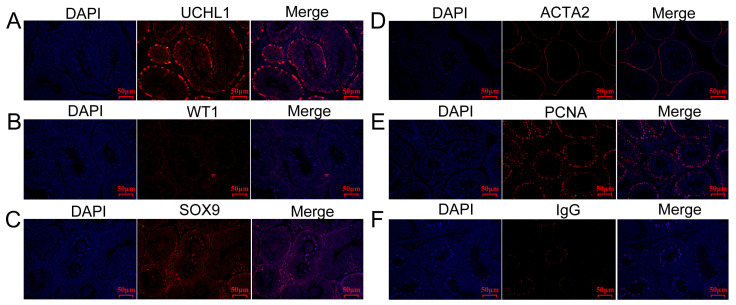
Localization of the protein products of several marker genes using section immunofluorescence staining. The distribution of UCHL1 (**A**), WT1 (**B**), SOX9 (**C**), ACTA2 (**D**), and PCNA (**E**) expression in Hezuo pig testicular cells. As a control (**F**), IgG was used instead of the primary antibody. Scale bar = 50 μm.

We next performed an immunohistochemical analysis on testicular tissue to characterize testicular cells and thus validate the expression patterns of several marker genes at the protein level. The results showed that UCHL1 was expressed in a circle of SPG adjacent to seminiferous tubules (Figure 9A), suggesting that UCHL1 may serve as a spermatogonial marker in the Hezuo pig. WT1 was expressed in SCs from the seminiferous lumen to seminiferous tubules (Figure 9B), and SOX9 was highly expressed in these cells (Figure 9C), suggesting that these two genes have the potential to serve as markers for the characterization of SCs in the Hezuo pig. α-Smooth muscle actin (α-SMA), encoded by *ACTA2*, is a widely used marker for MCs in human and mouse testes. In this study, MCs in Hezuo pig testes were also characterized by α-SMA staining (Figure 9D), indicating that ACTA2 is a conserved marker gene for MCs in mammalian species. PCNA is highly expressed in proliferating cells. As expected, in this study, PCNA was extensively expressed in germ cells and somatic cells (LCs, SPG, and SPCs) (Figure 9E), suggesting that these cells were actively proliferating. Control (IgG) staining is shown in Figure 9F.

## 3. Discussion

The testis is the most important male reproductive organ, and its structural and functional integrity is vital for sperm production [38,39,40]. In male animals, lifelong fertility depends on continuous spermatogenesis, and the interaction between germ cells and somatic cells is critical for the maintenance of this process in the testes [41,42]. Studies involving testicular cell transcriptome profiling are commonly based on the whole testicular tissue; accordingly, knowledge regarding germ and somatic cell-specific gene expression has remained limited [28,43]. To acquire testicular cell type-specific gene expression profiles, numerous studies have employed techniques such as STA-PUT, MACS, FACS, and LCM, which typically require large numbers of all cell populations [33]. ScRNA-seq has been increasingly used for this purpose owing to the advantages of requiring fewer cells and allowing deeper assessment of cell heterogeneity [25,44]. High-dimensional scRNA-seq approaches have provided an invaluable means of investigating the development and differentiation of both germ and somatic cell populations in the testes of mice, humans, and domestic animals such as cattle, yaks, sheep, goats, and chickens [23,45,46,47].

In this study, using the universal 10× Genomics platform, we utilized a scRNA-seq approach to explore the heterogeneity of testicular cells in the Hezuo pig and thus provide information regarding the dynamic and differential transcriptomes of testicular cells in this pig breed. We obtained a total of 16,082 cells from sexually mature Hezuo pig testes, and, based on known cell type-specific marker genes in mice, humans, and other mammalian species [19,33,35], we identified the main germ cell and somatic cell types (five somatic and four germ cell types). We also screened the DEGs in each cell type and conducted a functional enrichment analysis for the identified testicular cell types and investigated the localization of the protein products of some marker genes in the testes of Hezuo pigs using immunohistochemistry. 

LCs are located at the periphery of the seminiferous tubules, and their primary function is to produce testosterone, which is extremely important for the maintenance of spermatogenesis, male secondary sexual features, and systemic metabolism [48,49,50]. Meanwhile, MCs are located between seminiferous tubules, where they perform contractile functions and secrete glial cell-derived neurotrophic factor (GDNF), which is essential for spermatogenic processes [37]. Yu et al. performed a Monocle pseudo-time analysis on LCs and MCs of dairy goat testes and found that both cell types were differentiated from common progenitor cells [30]. Using a similar methodology, Wang et al. found that yak LCs and MCs were grouped in one large cluster and also shared common progenitors [19]. To reveal whether this phenomenon also exists in Hezuo pigs, we performed a cluster analysis of Hezuo pig testicular cells. Consistent with the results reported by Yu et al. [30] and Wang et al. [19], we found that LCs and MCs were grouped in a large cluster, while Monocle pseudo-time analysis further indicated that they originated from common progenitor cells.

In mammalian testes, spermatogenic development and continual spermatogenesis highly rely on various types of testicular somatic cells, including SCs, LCs, MCs, ECs, and immune cells [32,51]. Here, we observed that five somatic cell types, namely, SCs, LCs, MCs, ECs, and NK cells/macrophages, clearly clustered together. In the seminiferous tubules of the testis, different germ cells make direct contact only with SCs, which perform vital “nursing functions” for spermatogonial self-renewal, proliferation, and differentiation [52,53]. Additionally, SCs are indispensable for the maintenance of the normal function of LCs and MCs in the testis [54]. Meanwhile, testicular immune cells, such as NK cells and macrophages, participate in the mediation and regulation of immune responses, processes that are indispensable for the maintenance of testicular homeostasis [55]. The interplay between germ and somatic cells can safeguard normal and continuous spermatogenesis, thereby guaranteeing male fertility. Interestingly, in this study, we identified all these testicular somatic cell types, and they even occupied most sequenced cell populations. Moreover, the fact that we identified somatic cell types in the Hezuo pig using known cell marker genes from mice and humans is indicative of conserved gene expression patterns across mammalian testes. Such a possibility can be supported by GO functional enrichment and KEGG pathways analysis, which can validate the similarities in biological processes and functions related to these cell types across different mammalian species. Indeed, given the important functions of somatic cells, many studies have undertaken functional enrichment analyses of DEGs in testicular somatic cells of humans and several livestock animals. For example, Wang et al. found that the DEGs in testicular SCs, MCs, and LCs in yak were mainly enriched in the cAMP, estrogen, PI3K–Akt, MAPK, ECM–receptor interaction, and protein processing in the endoplasmic reticulum signaling pathways and also were associated with the GO terms cell part, metabolic process, and biological adhesion [19]. Zhang et al. showed that, in sheep testes, the DEGs in MCs were mainly involved in extracellular matrix organization and regulation of cell adhesion, LCs were enriched in testosterone synthesis, and SCs were enriched in regulation of cell death and exocytosis [33]. In a similar analysis of somatic cells in yak and porcine testes, Yang et al. demonstrated that SCs and LCs were enriched in the ribosomal pathway, which is associated with the function of protein molecules in sheep somatic cells [22]. To further explore the role of testicular somatic cells in Hezuo pig spermatogenesis, we carried out a functional enrichment analysis of the DEGs of five somatic cell types. We found that the DEGs in SCs were mainly enriched in the thyroid hormone and Wnt signaling pathways and were also involved in regulation of cellular component organization, cell part, protein binding, and enzyme binding. Meanwhile, the DEGs in LCs and MCs were mainly enriched in the PI3K–Akt, vascular smooth muscle contraction, steroid biosynthesis, and cortisol synthesis and secretion signaling pathways and were primarily associated with the GO terms organic substance metabolic process, intracellular organelle part, and basement membrane, indicating the participation of protein molecules in the function of Hezuo pig testicular somatic cells. The DEGs in NK cells/macrophages were involved in the antigen processing and presentation signaling pathway as well as immune response, activation of immune response, and response to cytokine, suggesting that immune cells play vital roles in resisting exogenous microorganisms and maintaining homeostasis in the testes of Hezuo pigs. Indeed, several KEGG signaling pathways and GO terms were similar to those reported in yak testis by Wang et al. [19] and in porcine testis by Zhang et al. [35]. Although the functional enrichment of DEGs in testicular somatic cells differed slightly among various species, the main function of the DEGs remains the assistance of the sperm production process.

In our study, we identified four male germ cell types (SPG, SPCs, SPTs, and SP cells) based on known marker genes. We did not identify other subtypes of SPG, which is in contrast to some reports [33,56] but consistent with the results reported for the yak [19]. This may be due to the small proportion of undifferentiated SPG in the Hezuo pig testis, which resulted in a limited number of cell types being identified. During spermatogenesis in Hezuo pigs, some genes, such as *SYCP1*, were continuously expressed throughout the whole spermatogenic process but were extremely highly expressed at specific stages. *SYCP1* is important for meiotic chromosome synapsis during spermatocyte development as well as for male fertility and is also involved in centromere pairing during meiosis and the assembly of the central element of synaptonemal complexes [57,58]. In this study, *SYCP1* was specifically expressed in SPCs. Moreover, we identified many specifically expressed genes that have not been defined or reported in the various cell types analyzed, such as *LOC106504743*, *LOC102166036*, and *LOC110260189*. These genes may play significant roles in Hezuo pig spermatogenesis. Our findings indicated that there may be numerous spermatogenesis-specific regulatory genes in Hezuo pigs, a possibility that requires in-depth annotation and investigation.

GO functional enrichment analysis revealed that the DEGs in SPG were related to protein binding, DNA binding, ATP binding, cell development, and hydrogen transport, which is consistent with that reported for goats [30] and yaks [19]. As expected, the DEGs in SPCs were enriched in terms such as nucleus, DNA binding, cell part, reproduction, and spermatogenesis. Additionally, the DEGs in SPTs and SP cells were mainly enriched in cytoskeleton, sexual reproduction, and multi-organism reproductive processes, in line with studies on yak [19] and buffalo [28] testes. Moreover, the DGEs in SPG were mainly enriched in the cell cycle signaling pathway, in keeping with the greater proliferative ability of SPG [59]. Indeed, DEGs associated with the cell cycle signaling pathway were also found to be enriched in the yak testis [19]. The DGEs in SPCs were enriched in both the cell cycle and PI3K–Akt signaling pathway, suggesting that these genes were mainly related to meiosis, which is consistent with studies on goat and buffalo testes [28]. Taken together, our results suggested that germ cells play vital roles in the reproduction process in Hezuo pigs, as determined by GO and KEGG functional analysis.

In this study, we screened DEGs in each cell cluster, and these DEGs may serve as important molecular markers for specific testicular cell types in the Hezuo pig. Then, using immunohistochemistry, we investigated the protein localization patterns for some marker genes, such as *UCHL1*, *WT1*, *SOX9*, *ACTA2*, and *PCNA*, for which commercially available antibodies worked in the Hezuo pig. As expected, UCHL1 characterized SPG in the Hezuo pig, in line with that reported for buffalo (Huang et al. [28]), giant panda (Zheng et al. [35]), and humans [37]. *WT1* plays an important role in cellular development and is a potential marker for SCs [35]. Indeed, *WT1* was extensively expressed in Hezuo pig SCs, as was also observed in the giant panda. *SOX9* is commonly used as a marker for identifying SCs in testes from all age groups [30,60]. In our study, SOX9 protein was found to localize in SCs of Hezuo pig testes. Additionally, in our study, peritubular MCs were characterized by α-SMA expression. *PCNA* is a cell proliferation marker for a wide variety of cell types [61,62]. Here, PCNA was found to be highly localized in proliferating somatic cells (SCs) and germ cells (SPG and SP cells), which is consistent with results reported for the giant panda [35]. Consequently, these results demonstrated that these genes may serve as molecular markers for specific cell types in the testes of the Hezuo pig, which will facilitate in-depth investigations on male reproduction in this breed.

To our knowledge, ours is the first study to provide a systematic classification of cell groups in the Hezuo pig testis based on previous studies on humans, mice, and sheep. We identified the main cell types, clarified the associated biological processes, and suggested marker genes for future studies on their functions. Additionally, we provided a detailed classification of Hezuo pig germ cells and cell surface markers for studying spermatogonial stem cell development in vitro. Our dataset and analysis are expected to offer valuable resources and references for studies of male Hezuo pig reproduction, as well as the long-term genetic improvement and preservation of this breed.

## 4. Materials and Methods

### 4.1. Ethics Statement

All animal-related work was conducted in accordance with the guidelines established by the Ministry of Agriculture in China. All Hezuo pig handling procedures were approved by the Committee on Animal Ethics of Gansu Agricultural University (Permit No. 2006-398). Every effort was made to minimize the suffering of the animals.

### 4.2. Testicular Sample Collection

The testes used for scRNA-seq were obtained from a 4-month-old healthy male Hezuo pig from Hezuo County in Gannan Tibetan Autonomous Prefecture. After collection, the testes were washed three times with pre-cooled Dulbecco’s phosphate-buffered saline (DPBS, Gibco, Waltham, MA, USA) to remove the testicular white membrane and epithelium. The testicular tissue was then cut into several small fragments and placed in cryotubes containing cryopreservation medium composed of 70% Dulbecco’s modified Eagle’s medium (DMEM, Invitrogen, Carlsbad, CA, USA), 20% fetal bovine serum (FBS, Gibco, Waltham, MA, USA), 8% dimethyl sulfoxide (DMSO, Sigma-Aldrich, St. Louis, MO, USA), and 2% penicillin–streptomycin (Gibco, Waltham, MA, USA), followed by slow freezing and storage in liquid nitrogen.

### 4.3. Testicular Single-Cell Suspension Acquisition

Single-cell suspensions were obtained by enzymatic digestion. Frozen testicular tissue blocks were thawed by immediately placing them in a 37 °C water bath for 3 min. The testicular fragments were then incubated with 1 mg/mL type IV collagenase (Invitrogen) for 8 min at 37 °C with shaking (100 rpm), washed twice with pre-cooled DPBS, and incubated with 0.25% trypsin-EDTA (Gibco, Waltham, MA, USA) and 0.25 mg/mL DNase I (Sigma-Aldrich, St. Louis, MO, USA) in an incubator at 37 °C for 10 min with 15 gentle pulses given every 3 min with a blunt tip. The digestion was stopped by adding an equal volume of DMEM containing 10% FBS. Single testicular cells were isolated by filtering through a cell strainer, centrifuged at 800× *g* for 5 min, and washed with pre-cooled DPBS. Afterward, the cells were counted using a Countess II Automated Cell Counter and resuspended in PBS containing 0.4% bovine serum albumin (Sigma-Aldrich, St. Louis, MO, USA) at a concentration appropriate for scRNA-seq.

### 4.4. 10× Genomics Library Preparation and Sequencing

An equal volume of 0.4% trypan blue staining solution was added to the single-cell suspensions for cell quality control, and the cell concentration was adjusted to 1500–2000 cells/mL. Then, the cell suspension was loaded onto a 10× Genomics GemCode Single-cell instrument (Pleasanton, CA, USA), and cell capture and library preparation were performed using the Chromium Next GEM Single Cell 30 Reagent Kit (v.3.1). The final library was sequenced at Guangzhou Genedenovo Biotechnology Co., Ltd. (Guangzhou, Guangdong, China) using the PE150 mode on an Illumina sequencing platform (NovaSeq 6000).

### 4.5. Genome Alignment and Gene Expression Quantification

Cell Ranger (v.3.1.0) software from 10× Genomics was used for sample quality control as well as to convert the raw BCL files to FASTQ files for alignment and count quantification. Then, reads with low-quality barcodes and UMIs were removed, and the remaining reads were then mapped to the *Sus scrofa* reference genome. Only reads uniquely mapped to the transcriptome and having at least 50% of their sequence intersecting an exon were considered for UMI counting. Before quantification, the UMI sequences were corrected for errors, and valid barcodes were identified based on the EmptyDrops method [63]. Finally, cell-by-gene matrices were obtained by UMI counting and cell barcode calling.

### 4.6. Cell Clustering

Seurat (v.3.1.1) software [64] was used to process the cell-by-gene matrices for downstream sample analysis. First, the following three inclusion criteria were applied: (i) percentage of expressed mitochondrial gene <10%; (ii) number of genes that could be detected per cell ≥200; and (iii) each gene had to be expressed in at least three cells. This was followed by data normalization, dimensionality reduction, clustering, and differential expression analysis. For clustering analysis, highly variable genes were identified, and the principal components predicted for these genes were used to construct a graph. t-SNE was used for dimensionality reduction clustering analysis and visualizing the Principal Component Analysis (PCA)-based cell clustering results. Eventually, the optimal cell clusters were obtained and were identified based on reported marker genes.

### 4.7. DEG Analysis and Annotation

To acquire DEGs in each cell cluster, differential expression analysis was performed using the Wilcoxon rank-sum test [65] based on the filtered gene expression matrices generated by Seurat [64]. Significantly upregulated genes were screened based on the following criteria: Genes had to be at least 1.28-fold overexpressed in the target cluster; genes had to be expressed in more than 25% of the cells belonging to the target cluster; and the Wilcoxon rank-sum test *p*-value had to be lower than 0.05.

GO and KEGG functional enrichment analyses of marker genes within each cluster were performed in the GOseq R package [66] and the KOBAS 3.0 package [67], respectively.

### 4.8. Cell Trajectory Analysis

Cell trajectory analysis (pseudo-time analysis) was used to investigate the differentiation trajectory of cells or the evolution of cell subtypes. The approach involves sorting cells at pseudo-time based on the expression patterns of key genes, which allows for the simulation of dynamic changes occurring during the development process. In this study, pseudo-time analysis of LCs and MCs was performed using the Monocle 2 (v.2.8.0) package [68].

### 4.9. Hematoxylin and Eosin (H&E) Staining and Immunohistochemical Analysis

Testicular tissue from a Hezuo pig was cut into small fragments, fixed in diluted Bouin’s solution for 72 h, embedded in paraffin, cut into 5 µm thick sections, deparaffinized, rehydrated, and stained with H&E for morphological observation (Servicebio, Wuhan, China). For immunohistochemical analysis, testis sections were subjected to heat-mediated antigen retrieval in Tris-EDTA buffer (pH 9.0) and then blocked with Super Block at room temperature for 1 h. This was followed by incubation first with primary antibodies at 4 °C overnight and then with appropriately diluted fluorescent secondary antibodies for 1 h at room temperature, avoiding light. The primary antibodies were mouse anti-UCHL1 (1:200, GB12159), rabbit anti-WT1 (1:200, GB11382), rabbit anti-SOX9 (1:200, GB11280), rabbit anti-ACTA2 (1:500, GB111364), and mouse anti-PCNA (1:500, GB12010), all purchased from Servicebio. Anti-rabbit IgG (1:200, GB111738, instead of the corresponding primary antibody) was used as the negative control. The secondary antibody was CY3-conjugated goat anti-rabbit IgG; DAPI was used for counterstaining.

## 5. Conclusions

In summary, we identified nine distinct cell types in Hezuo pig testis based on marker genes using scRNA-seq. Four were germ cells (SPG, SPCs, SPTs, and SP cells), and five were somatic cells (SCs, LCs, MCs, ECs, NK cells/macrophages). The DGEs for each cell type were identified and subjected to functional enrichment analysis. Our findings indicated that Hezuo pig LCs and MCs originated from common progenitor cells. Moreover, we identified *UCHL1* as a marker gene for SPG, *WT1* and *SOX9* as marker genes for SCs, and *ACTA2* as a marker gene for MCs in Hezuo pig testis using immunohistochemistry. Our dataset provides valuable insights into Hezuo pig spermatogenesis and paves the way for identifying key molecular markers involved in male germ cell development.

## Data Availability

Data are contained within the article and Supplementary Materials.

## Supplementary Materials

The following supporting information can be downloaded at: https://www.mdpi.com/article/10.3390/ijms25189786/s1.
