# Supplementary material for: Single-Cell RNA Sequencing Reveals an Atlas of Hezuo Pig Testis Cells
Source: Int J Mol Sci. 2024 Sep 10;25(18):9786. doi: 10.3390/ijms25189786 (PMC11431743; doi:10.3390/ijms25189786)
Supplement: Supplementary file 1 [file ijms-25-09786-s001.zip › Supplementary Materials.pdf]

Supplementary Materials

Figure S1. Violin plots of the selected marker genes in the different clusters.

Figure S2. The expression of 32 cell-specific genes in porcine testicular cells.

Figure S3. A heatmap of 32 cell-specific genes in porcine testicular cells.

Figure S4. Feature plots (t-distributed stochastic neighbor embedding [t-SNE]) of 32 cell-specific marker genes in each cluster.

Figure S5. Gene Ontology (GO) and Kyoto Encyclopedia of Genes and Genomes (KEGG) enrichment analysis for myoid cells (MCs), endothelial cells (ECs), and natural killer (NK) cells/macrophages.

Figure S6. Kyoto Encyclopedia of Genes and Genomes (KEGG) enrichment analysis for spermatogonia (SPG), spermatocytes (SPCs), spermatids (SPTs), and sperm (SP) cells.

Table S1. Information for all the single cells.

Table S2. Metrics summary.

Table S3. Functional enrichment analysis of Sertoli cells (SCs).

Table S4. Functional enrichment analysis of Leydig cells (LCs).

Table S5. Functional enrichment analysis of myoid cells (MCs). Table S6: Functional enrichment analysis of endothelial cells (ECs).

Table S7. Functional enrichment analysis of natural killer (NK) cells/macrophages.

Table S8. Functional enrichment analysis of spermatogonia (SPG).

Table S9. Functional enrichment analysis of spermatocytes (SPCs). Table S10: Functional enrichment analysis of spermatids (SPTs). Table S11: Functional enrichment analysis of sperm (SP) cells.
