# Supplementary figures and images for: Single-Cell RNA Sequencing Reveals an Atlas of Hezuo Pig Testis Cells
Source: Int J Mol Sci. 2024 Sep 10;25(18):9786. doi: 10.3390/ijms25189786 (PMC11431743; doi:10.3390/ijms25189786)

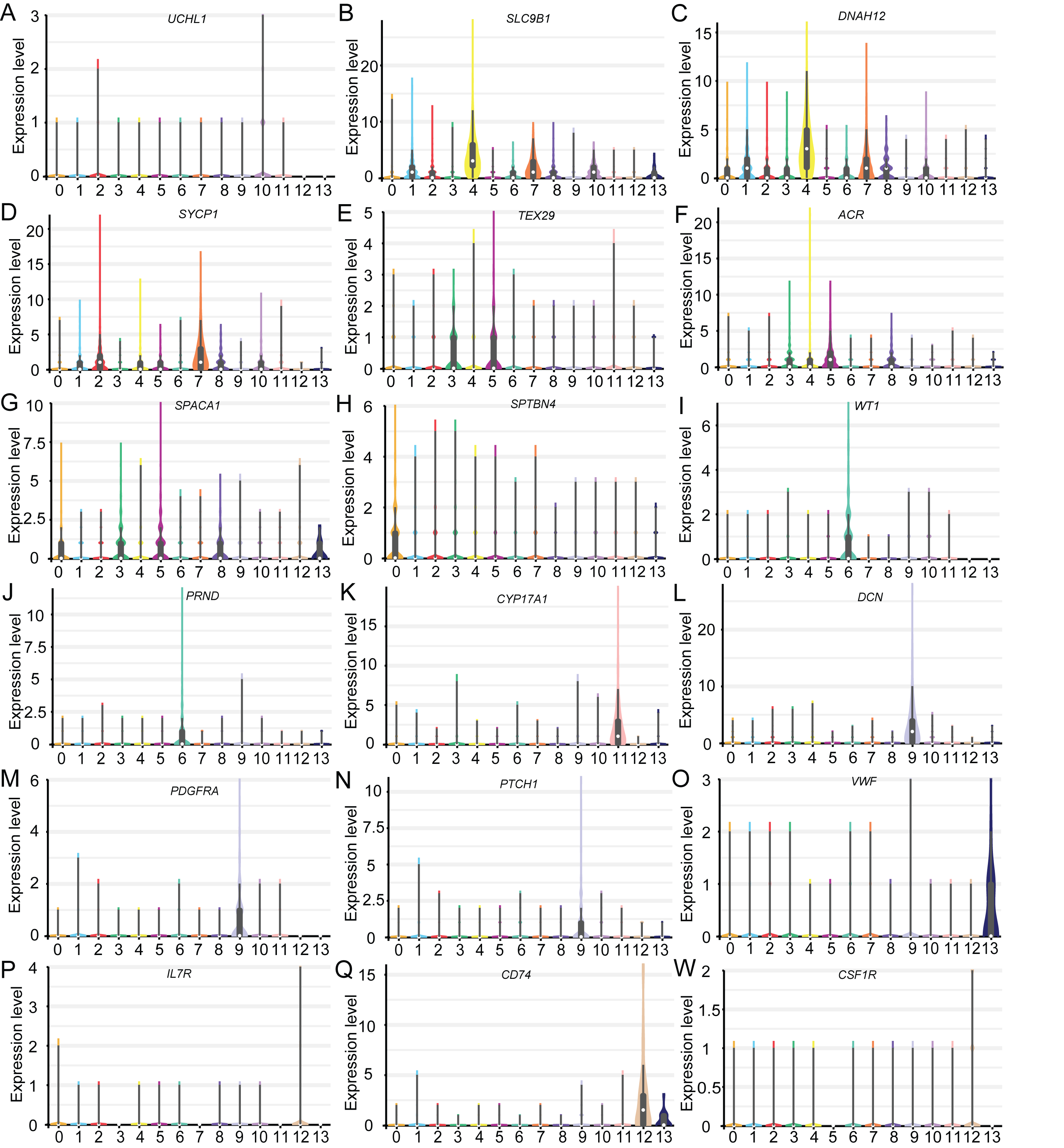

Supplement: Supplementary file 1 [file ijms-25-09786-s001.zip › Figure S1.tif]

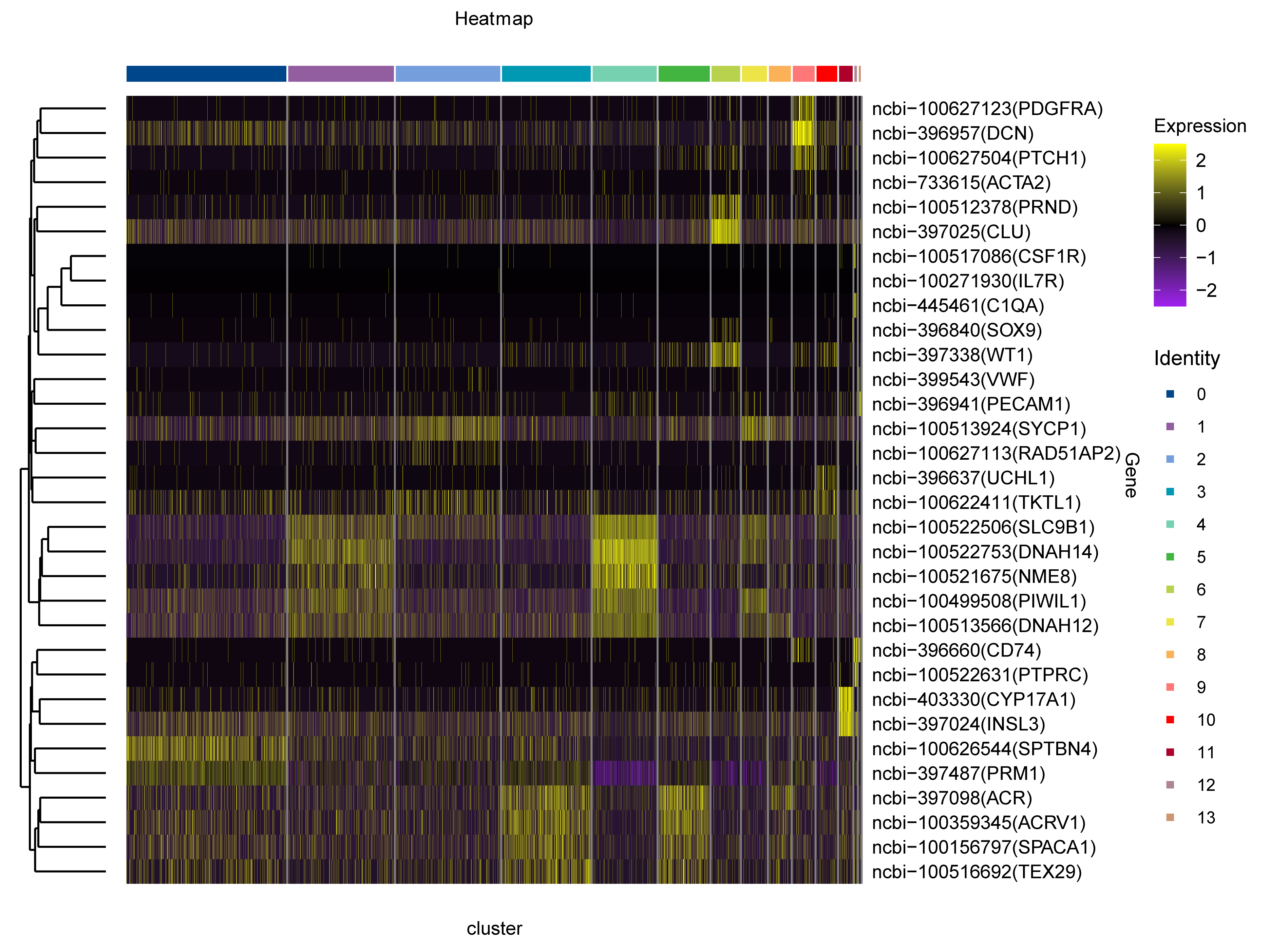

Supplement: Supplementary file 1 [file ijms-25-09786-s001.zip › Figure S3.tif]

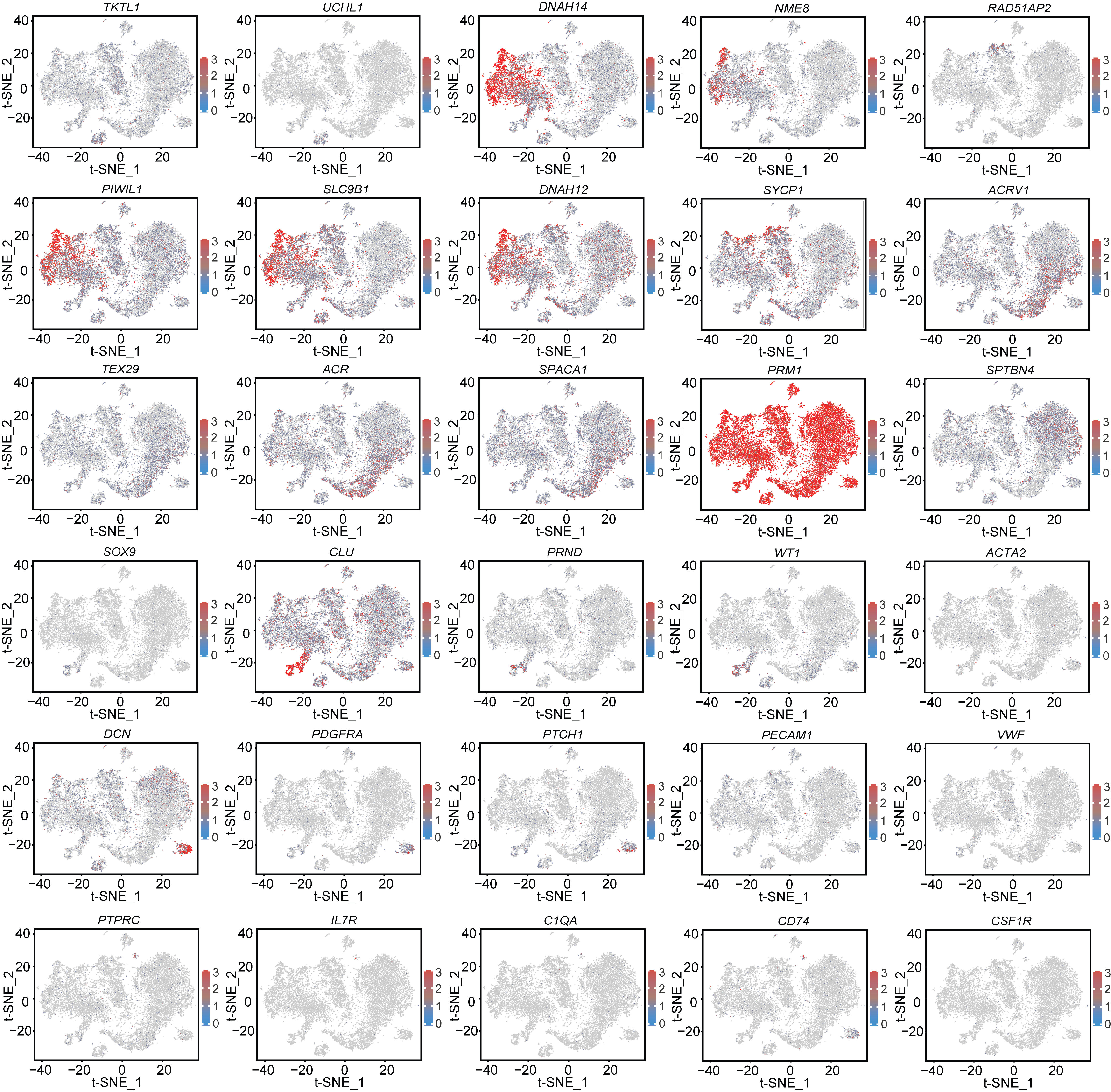

Supplement: Supplementary file 1 [file ijms-25-09786-s001.zip › Figure S4.tif]

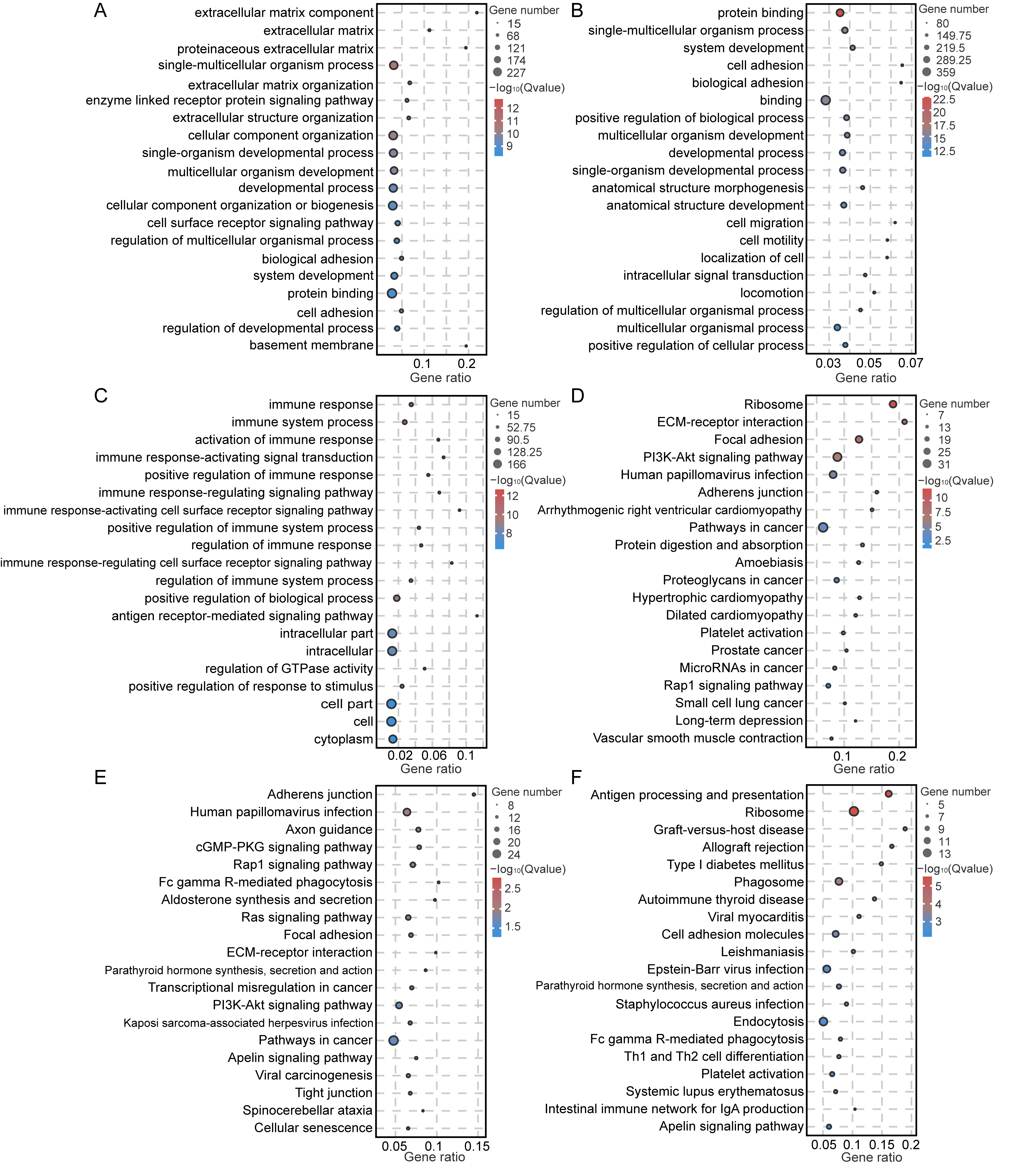

Supplement: Supplementary file 1 [file ijms-25-09786-s001.zip › Figure S5.tif]

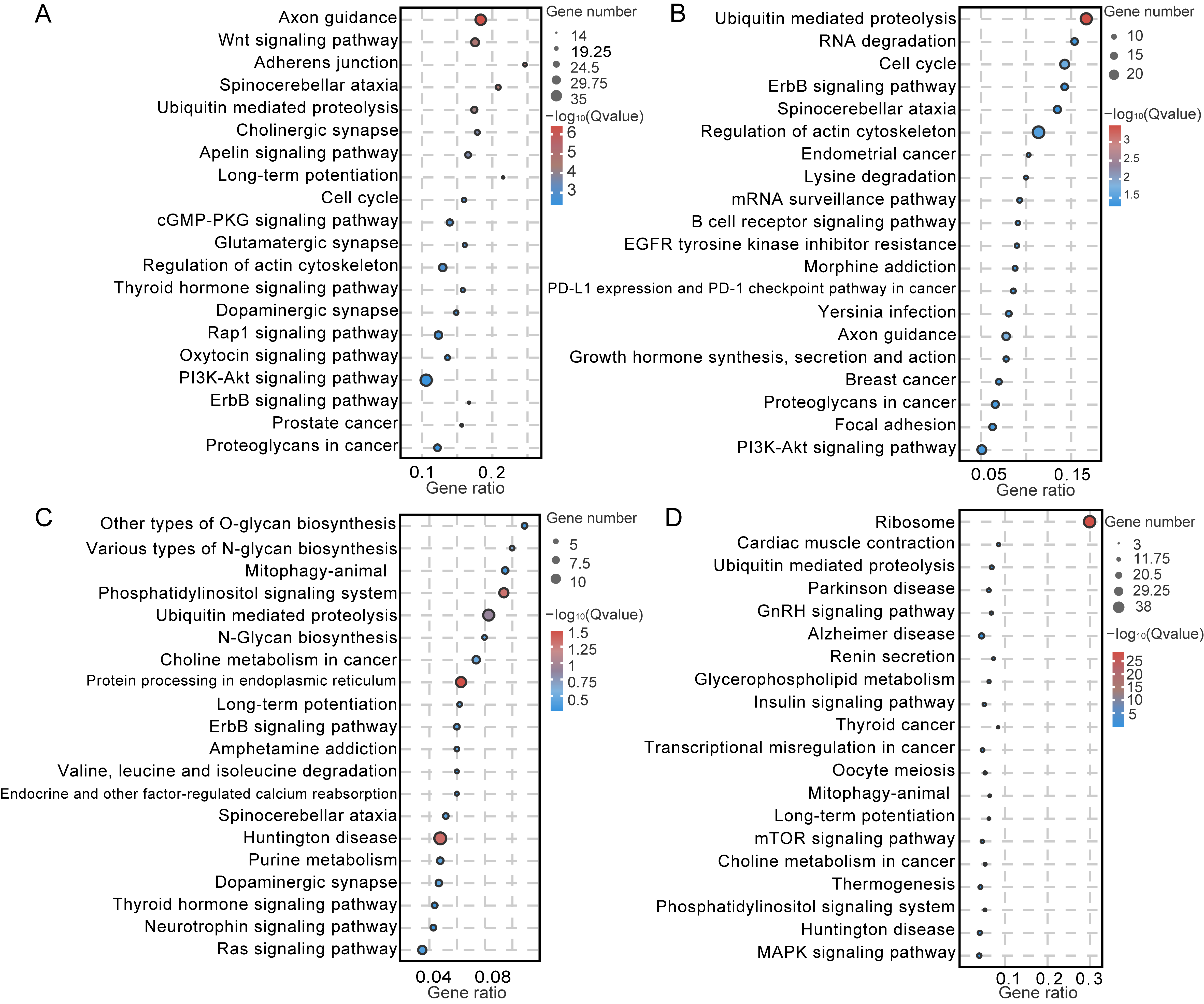

Supplement: Supplementary file 1 [file ijms-25-09786-s001.zip › Figure S6.tif]
